# Progressive multiple sclerosis: Evaluating current therapies and exploring future treatment strategies

**DOI:** 10.1016/j.neurot.2025.e00601

**Published:** 2025-05-09

**Authors:** Marelisa Albelo-Martínez, Syed Rizvi

**Affiliations:** Department of Neurology, Brown University Health and Rhode Island Hospital, Alpert Medical School of Brown University, USA

**Keywords:** Progressive multiple sclerosis, Secondary progressive multiple sclerosis, Primary progressive multiple sclerosis, Disease modifying therapy, Progression independent of disease activity, Smoldering inflammation

## Abstract

Progressive forms of multiple sclerosis (MS) include primary progressive MS (PPMS) and secondary progressive MS (SPMS). Unlike relapsing-remitting MS (RRMS), progressive MS is recognized by relentless progression with accumulating disability, rare to no relapses nor new activity on MRIs. Clinically, neurologic worsening in MS can occur in the relapsing-remitting (RRMS) phase of disease due to incomplete recovery from neuroinflammatory relapses. However, a progressive disease course is the dominant factor related to accumulating disability. There is persistent central nervous system (CNS) compartmentalized inflammation, mitochondrial dysfunction and altered immune responses. Unlike in RRMS, the efficacy of disease modifying agents (DMA) in progressive MS has been limited, highlighting the need for novel therapeutic approaches that address both inflammation and neurodegeneration. This article explores current management of progressive MS, and future directions in targeting the unique pathophysiology of this complex disease.

## Introduction

Multiple Sclerosis is a chronic, immune-mediated degenerative disorder of the CNS characterized by demyelination, axonal injury, gliosis, and progressive brain atrophy [1]. RRMS constitutes approximately 85 ​% of initial diagnoses and is characterized by acute episodes followed by periods of remission and stability [2]. SPMS typically evolves from RRMS with a gradual accumulation of neurological disability and reduced relapse activity [2]. Primary Progressive MS, seen in 10–15 ​% of patients is characterized by continuous neurological decline from disease onset with very rare distinct relapses [3]. Both SPMS and PPMS are included in the category of progressive MS (PMS).

## Diagnosis

The diagnostic criteria for PPMS require at least one year of continuous neurological symptom progression, along with evidence of CNS demyelination in at least two areas (brain or spinal cord) on magnetic resonance imaging (MRI) or positive cerebrospinal fluid (CSF) findings (e.g., oligoclonal bands) [4]. Additionally, other possible causes of progressive neurological decline must be excluded. The therapeutic landscape and understanding of SPMS have evolved, but standardized diagnostic criteria or biomarkers for SPMS remain lacking. SPMS is often diagnosed retrospectively after 3–12 months of neurological impairment independent of relapses [5]. There have been various proposed criteria but one is not accepted over the others. For example, the Lorscheider criteria [6] derived from the MSBase cohort, suggest progression of disability independent of relapses with an Expanded Disability Status Scale (EDSS) of ≥4.0. The 2014 Lublin criteria [7] further classify SPMS into active and non-active forms, based on clinical and MRI activity.

Despite the traditional classification of multiple sclerosis into progressive and relapsing-remitting forms, recent studies indicate that all types of MS are progressive from the start, even throughout the so-called “relapsing-remitting” stages. Studies demonstrating that neurodegeneration, characterized by axonal loss, brain atrophy, and the progressive accumulation of disability, starts in the early stages of the illness [8,9] lend credence to this theory.

## Pathophysiology

Recent studies have demonstrated that components of inflammation and neurodegeneration are present in all MS phases [8]. However, features of inflammation are more prominent during the relapsing stage and the neurodegenerative component predominates in the progressive stages [9]. Although both aspects exist in parallel since disease onset, MS becomes clinically progressive when the magnitude of the injury surpasses the CNS’s ability to functionally compensate, leading to continuous neurological disability [10].

Whereas relapsing MS is driven by acute inflammation associated with disruption of the blood-brain barrier, in progressive MS the immune response occurs within the CNS without disruption of the blood-brain barrier [11]. This localized and compartmentalized inflammation consists of lymphocytic infiltrates of T and B lymphocytes, plasma cells, and macrophages that form lymphoid follicles in the meninges and the perivascular spaces [9]. In addition, in progressive MS there is profound microglial activation related to oxidative stress, resulting in mitochondrial injury [9]. Both the meningeal inflammation and oxidative stress have been implicated in the marked axonal degeneration that characterizes progressive forms of MS [12]. Degenerating axons have been found throughout the whole white matter in MS patients, and patients with progressive disease show significantly more diffuse axonal injury in the normal appearing white matter [13].

Diffuse white matter injury and slow expansion of pre-existing white matter lesions (“smoldering” or “slowly expanding lesions”) are hallmark characteristics of progressive disease [10]. Nevertheless, grey matter is also heavily affected in progressive MS. Prominent meningeal-associated cortical lesions and subpial lesions are thought to be one of the driving forces behind progression independent of relapse (PIRA) [14,15]. These slowly expanding lesions, also knowns as chronic active-inactive lesions, are characterized by a rim of microglia, lymphocytes, and macrophages around an inactive lesion have also been implicated in PIRA and have been proven to correlate with increased disability [16]. Paramagnetic rim lesions on MRI, indicative of chronic active lesions with ongoing inflammation, are seen in all forms of MS but reflect slow, progressive damage [17].

**Fig. 1:**
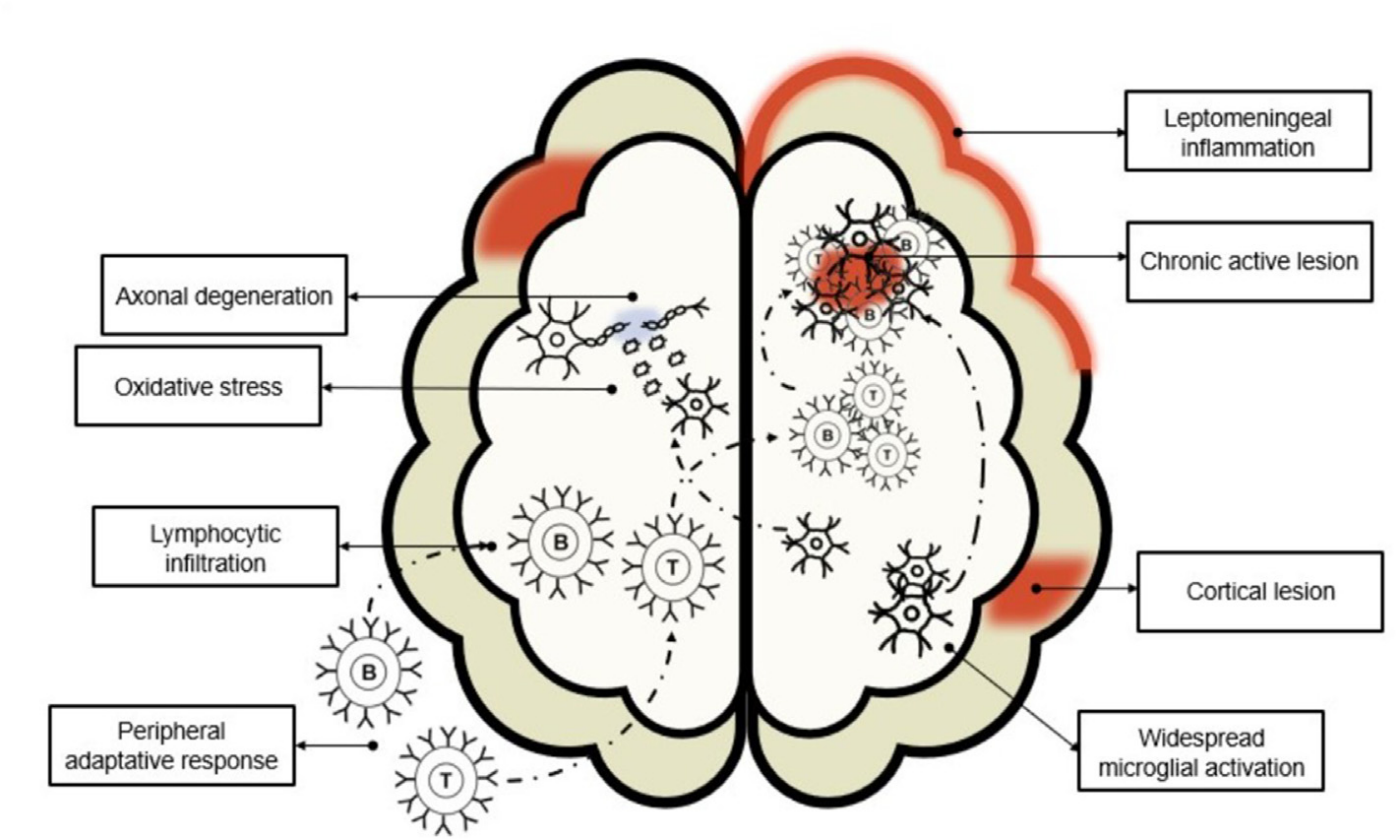
Shows the lymphocytic infiltration from the periphery to the CNS that is most prominent in relapsing MS. Also seen are characteristic features of progressive MS such as microglial activation, leptomeningeal inflammation, cortical lesions, axonal degeneration caused by oxidate stress, and a chronic active lesion which is surrounded by lymphocytes and activated microglia.

In PPMS, the disease is typically driven by less overt inflammation but more diffuse CNS damage, with axonal degeneration and gliosis playing prominent roles in pathophysiology [18]. SPMS, on the other hand, evolves from RRMS usually within 10–20 years after the initial diagnosis. This transition is marked by a shift from the relapsing-remitting pattern to a steady progression of disability, with or without superimposed relapses. In SPMS, the inflammatory activity seen in RRMS diminishes over time, while neurodegenerative processes become more prominent [9]. Some of these pathophysiologic mechanisms are illustrated in Fig. 1 and summarized in Table 1.

**Table 1:** Pathological features of RRMS and Progressive MS.

Pathological Aspect	RRMS	Progressive MS [PPMS, SPMS]

Inflammatory activity	High, episodic inflammation; relapses with remission.	Chronic, low-grade inflammation; includes ongoing inflammation at lesion edges in smoldering MS, with fewer relapses.
Lesion distribution	Discrete, well-defined lesions, often perivenular.	Widespread demyelination with cortical involvement; expanding “smoldering” lesions common in progressive MS.
Paramagnetic lesions (iron-laden)	Rarely observed, more typical in acute lesions.	Prominent in smoldering MS; also known as slowly expanding lesions. Appear as iron-laden, chronically active lesions with paramagnetic “rings” on MRI.
Myelin damage	Prominent during relapses with partial remyelination potential.	Extensive and progressive myelin loss; smoldering lesions show continuous demyelination at lesion edges.
Axonal damage	Mild to moderate, primarily associated with relapses.	Significant, ongoing axonal loss in smoldering lesions, leading to cumulative disability.
Gray matter involvement	Primarily white matter lesions with some cortical impact.	Significant cortical demyelination and gray matter atrophy, especially in progressive stages.
Microglial activation	High during active lesions, more localized.	Persistent microglial activation in smoldering MS, especially at lesion edges. Chronic activation seen in both gray and white matter.
Mitochondrial dysfunction	Less prominent, mostly related to relapses.	Persistent in progressive MS, especially in smoldering lesions, contributing to neurodegeneration.
Neurodegeneration	Less pronounced, primarily relapse driven.	Ongoing, with progressive neurodegeneration in gray and white matter; smoldering lesions contribute to continuous neurodegenerative activity.
Blood-brain barrier (BBB) leakage	Frequent during relapses; enhancement on MRI.	Inflammation is more compartmentalized within the CNS with less BBB leakage. Less frequent enhancement in MRI
B-cell infiltration	Transient during active relapses.	Persistent B-cell presence, especially in SPMS, with B-cell follicles in meninges; linked to chronic inflammation in smoldering lesions.
Remyelination potential	Present, especially after relapses.	Limited; smoldering lesions have ongoing low-grade inflammation which impedes remyelination, with impaired OPC differentiation in progressive MS.
Disability progression	Stepwise, often relapse-related with periods of recovery.	Gradual, steady accumulation of disability; smoldering lesions contribute to slow, continuous worsening without recovery.

**Progression Independent of Relapse Activity (PIRA)** refers to the gradual worsening of disability in MS that occurs without any relapses, highlighting a mechanism of disease progression distinct from inflammatory attacks [19]. Although this concept is not yet well studied and not all PIRA is sustained, data derived from randomized clinical trials showed that PIRA events comprised approximately 70 ​%–90 ​% of all disability accrual events in a follow-up time of 2–10 years [10]. The pathophysiology of PIRA is believed to be driven by chronic neurodegeneration, involving mechanisms like microglial activation, oxidative stress, mitochondrial dysfunction, and slow axonal loss, rather than acute inflammatory demyelination [15]. Several trials have provided insights into PIRA. In the ORATORIO trial [20], Ocrelizumab showed efficacy in reducing disability progression in PPMS. Similarly, in the EXPAND trial [21], Siponimod demonstrated benefits in reducing progression in SPMS by modulating sphingosine-1-phosphate receptors, reducing neuroinflammation and preserving neurons. Additionally, OPERA trials [22] demonstrated the ability of Ocrelizumab to reduce PIRA-related disability progression in RRMS. PIRA starts early in the disease process and occurs in all phenotypes of MS [23]. PIRA represents a critical challenge in MS treatment, as traditional therapies focused on relapse prevention often have limited efficacy in halting disability accumulation in PIRA. Understanding and targeting PIRA is now seen as essential for addressing the full spectrum of MS progression.

## Symptom management

Symptom management in progressive forms of MS primarily aims to enhance quality of life by addressing specific symptoms as the disease advances. Common symptoms include spasticity, fatigue, pain, bladder and bowel dysfunction, and cognitive impairment [24]. Treatment strategies typically involve a combination of pharmacological therapies, physical rehabilitation, and lifestyle modifications. Pharmacological interventions (Table 2) target symptom relief while non-pharmacological therapies such as physical therapy, occupational therapy, and cognitive training play critical roles in improving and maintaining daily functioning.

**Table 2:** Drugs used for symptom management in progressive MS.

Symptom	Medications	Notes

Spasticity	Baclofen, tizanidine, diazepam, dantrolene, botulinum toxin	Consider adding physical therapy.
Fatigue	Amantadine, modafinil, adderall, methylphenidate	CNS stimulants may alleviate MS-related fatigue.
Pain	Gabapentin, pregabalin, duloxetine, carbamazepine	Anticonvulsants or antidepressants for neuropathic pain.
Bladder dysfunction	Oxybutynin, tolterodine, mirabegron	Anticholinergics or beta-3 agonists for overactive bladder.
Bowel dysfunction	Laxatives, stool softeners (e.g., polyethylene glycol)	Used to manage constipation, fiber supplements may also be beneficial.
Cognitive impairment	Donepezil, rivastigmine	Cholinesterase inhibitors, although evidence is limited.
Depression	SSRIs (e.g. sertraline, fluoxetine, citalopram, etc), SNRIs, (e.g. duloxetine, venlafaxine) bupropion, mirtazapine, trazodone	Antidepressants are commonly used in MS patients.
Gait abnormality	Dalfampridine	Used to improve walking.

## Physical therapy and rehabilitation

Physical therapy (PT) and rehabilitation are essential for managing symptoms and improving the quality of life for individuals with progressive MS, with substantial evidence supporting their efficacy. Research has shown that PT interventions, such as task-specific training, balance exercises, and gait training, significantly improve mobility and reduce fall risk in patients with progressive MS [25,26]. PT also helps alleviate muscle spasticity and manage fatigue, which are common debilitating symptoms of the disease [27,28]. Studies have demonstrated that aerobic exercises and resistance training, integral components of PT, led to significant reductions in fatigue levels among individuals with progressive MS [28]. These findings highlight the importance of PT and rehabilitation in addressing mobility impairments, spasticity, and fatigue, while also promoting functional independence and emotional well-being.

## Lifestyle modifications and supportive therapies

Lifestyle modifications play a crucial role in managing progressive MS. Vitamin D supplementation is recommended although studies have shown mixed results. A meta-analysis suggested that higher vitamin D levels are associated with a reduced risk of MS disease activity [29]. A more recent double-blind, placebo-controlled study found that oral cholecalciferol (100,000 IU every two weeks) significantly reduced disease activity in patients with clinically isolated syndrome (CIS) [30].

Regular exercise, including aerobic activities, strength training, and stretching, has been shown to improve fitness, mobility, and reduce fatigue in individuals with progressive MS. Exercise training has been shown to improve walking performance and reduce fatigue [31]. Stress management techniques, such as mindfulness meditation, yoga, and cognitive behavioral therapy (CBT), are also effective in reducing stress and improving quality of life [32].

Quitting smoking was associated with slower disability progression in MS patients [33]. Supportive therapies, such as occupational therapy (OT), speech and language therapy, and psychological support, are essential for maintaining independence and addressing the mental health challenges associated with MS [[34], [35], [36]]. Internet-based CBT self-management has emerged as a promising and cost-effective intervention for treating MS-related fatigue and improving broader outcomes like distress [37].

## Disease modifying treatments

The treatment options for progressive forms of multiple sclerosis have historically been limited. The development of disease-modifying therapies (DMTs) has been focused primarily on relapsing forms of MS, with fewer advances in progressive MS. The benefits of DMTs in progressive MS trials are often tied to the presence of inflammatory activity, such as relapses or new MRI lesions. DMTs target inflammation, so patients with active disease see more benefit, as the therapies help reduce relapses and lesion formation. In contrast, current DMTs are less effective in non-active progressive MS, where neurodegeneration drives disability [38]. While efficacy in reducing relapses in relapsing-remitting MS might be extrapolated to SPMS, extrapolating effects on disability progression would be inappropriate due to the differing pathophysiology between RMS and SPMS.

### Primary progressive MS

Ocrelizumab, a humanized monoclonal antibody that targets CD20-expressing B cells, was the first approved DMT for PPMS. It was evaluated in the ORATORIO trial [20] after earlier studies with rituximab showed limited efficacy in PPMS [39]. In the ORATORIO trial [20], 732 patients from ages 18 to 55 with maximum EDDS of 6.5 were randomized (2:1) to receive either ocrelizumab or placebo over three years. Ocrelizumab reduced the patients reaching 12-week confirmed disability progression by 24 ​% [HR ​= ​0.76]. Additionally, 42.7 ​% of Ocrelizumab-treated patients showed no evidence of progression, compared to 29.1 ​% in the placebo group.

In another multicenter, observational study OPPORTUNITY [40] ocrelizumab administration showed similar effects on disability progression in patients not meeting the ORATORIO eligibility criteria. Notably, this study included patients older than 55 years. Interestingly, there was no difference in outcome in patients with or without disease activity at the time on onset of treatment. This was also seen in an analysis of ORATARIO database [41].

Several other clinical trials have evaluated therapies for PPMS, with mixed results. Interferons and Glatiramer failed to significantly slow disability progression, showing little difference from placebo [[42], [43], [44]]. Fingolimod and Natalizumab both also failed to meet their primary endpoints in the INFORMS [45] and ASCEND [46] trials respectively, although Natalizumab showed some benefit in arm function. Rituximab did not meet its primary endpoint in the OLYMPUS trial [39] but showed delayed progression in younger patients. Masitinib, in a Phase III trial [47] significantly reduced the number of patients reaching disability progression, particularly in non-relapsing patients.

### Secondary progressive MS

Siponimod, a selective sphingosine-1-phosphate (S1P) receptor modulator, was studied in the EXPAND trial [21], which included patients with active SPMS. The trial demonstrated a 21 ​% reduction in disability progression compared to placebo. Notably, Siponimod improved cognitive processing speed, a key aspect of quality of life for SPMS patients. Post-hoc analyses revealed a 28 ​% reduction in sustained cognitive worsening and a 51 ​% increase in cognitive improvement among Siponimod-treated patients [48]. Ozanimod, another S1P modulator, was compared to teriflunomide in a study using matching-adjusted indirect comparison which showed modest improvements in confirmed disability progression (CDP) at 3 months, although without sustained effects at 6 months [49].

The Interferons were tested in several trials for SPMS. While the European study group of the multicentre randomised trial on Interferon-β-1b in SPMS [50], showed delayed timing to reaching CDP and reduced lesion volume, the North American study group did not show benefits. Subsequent meta-analyses revealed IFN-β’s limited efficacy in preventing disability progression, despite reducing relapse rates. Additional trials, including the SPECTRIMS [42] and IMPACT [51] studies, failed to confirm substantial benefits.

Mitoxantrone, a chemotherapeutic agent with immunosuppressive properties, has been used for treating SPMS due to its ability to reduce immune activity that drives the disease. In the MIMS trial [52], mitoxantrone showed efficacy in slowing disability progression and reducing relapse rates in SPMS patients. However, mitoxantrone use is limited by serious side effects, including cardiotoxicity and an increased risk of leukemia, for which it is currently very rarely used for MS.

Dimethyl fumarate and diroximel fumarate, both approved for relapsing forms of MS (including active SPMS), are thought to exert their effects by activating the nuclear factor erythroid 2-related factor 2 (Nrf2) pathway, which promotes anti-inflammatory and antioxidant responses. They shift T-cell profiles toward an anti-inflammatory state, reducing neuroinflammation. The DEFINE trial [53] confirmed a positive outcome in RRMS patients and demonstrated a 38 ​% reduction in 3-month CDP with dimethyl fumarate, although the efficacy of diroximel fumarate in SPMS remains uncertain.

Teriflunomide, a pyrimidine synthesis inhibitor, reduces the proliferation of activated T and B lymphocytes by inhibiting dihydroorotate dehydrogenase, a key enzyme in pyrimidine synthesis. It is approved for relapsing forms of MS and demonstrated a 31 ​% reduction in 3-month disability progression in the TEMSO trial [54], though only a small proportion of the study population had secondary progressive disease.

Cladribine is a deoxyadenosine analogue prodrug that preferentially depletes lymphocytes. The CLARITY study [55] reported a 33 ​% reduction in the risk of 3-month sustained disability progression compared to placebo, with post-hoc analyses suggesting that cladribine significantly reduced the risk of conversion to SPMS [56].

Natalizumab, a humanized monoclonal antibody targeting alpha 4-integrin is approved for RRMS and was evaluated for efficacy in SPMS in the ASCEND study [46]. The study found no significant effect of natalizumab on confirmed disability progression. However, natalizumab was found to be highly effective in reducing disability worsening in RRMS, as demonstrated in the AFFIRM trial [57].

Ofatumumab, like ocrelizumab, is a CD20-targeting antibody without known direct central nervous system effects. Although no specific studies for SPMS have been conducted, its efficacy and safety were evaluated in the ASCLEPIOS I and II trials [58], which included relapsing MS patients. In a pooled analysis of 108 patients with active SPMS, ofatumumab reduced ARR by 43 ​% and the risk of 6-month confirmed disability progression by 44 ​%, though these results had wide confidence intervals and were based on a small SPMS group [58].

Ocrelizumab is a monoclonal antibody targeting CD20^+^ cells, which are primarily B cells but also include a subset of CD3^+^ T cells. While it is not known to directly target the central nervous system, ocrelizumab has demonstrated efficacy in reducing disability progression in PPMS, as shown in the ORATORIO trial [20], which reported a 24 ​% risk reduction in disability progression at 24 weeks. Although no specific studies have been conducted in SPMS, post-hoc analyses of the OPERA trials [22], which focused on relapsing MS, suggest that ocrelizumab may also benefit patients with characteristics of SPMS. These analyses identified potential SPMS patients based on criteria like baseline EDSS ≥4.0 and pyramidal Functional System Score (FSS) ≥2, showing a 22–24 ​% reduction in relapse-independent disability progression.

Simvastatin has been evaluated in recent years as a possible treatment for non-active SPMS. The MS-STAT trial showed promising results in early phases with regards to brain atrophy [59]. However, the preliminary results of the Phase 3 trial discussed at ECTRIMS 2024 failed to show significant differences vs placebo [60].

In summary, only two DMTs have shown clear benefits in reducing disability progression, specifically in SPMS patients with activity. Siponimod, tested in the EXPAND study [21], and interferon-beta-1b in early active SPMS patients, as shown in the European Study [50]. Both treatments also demonstrated efficacy in preventing relapses. Notably, the population in the EXPAND study most closely mirrors clinical practice, where about 30 ​% of SPMS patients exhibit active disease. While other DMTs have been tested in small SPMS subpopulations within relapsing MS studies, the evidence remains vague, largely extrapolated from relapse reduction data in RRMS. RMS studies are underpowered to provide meaningful data on SPMS, particularly in terms of cognitive decline and progression independent of relapses. Moving forward, SPMS-specific trials should be prioritized, especially for addressing non-inflammatory disease progression, and more suitable clinical outcomes and progression measures need to be developed for future studies.

The best current strategy to prevent progression and the transition from RRMS to SPMS is to treat RRMS with highly effective DMTs from the onset of the disease. Early in MS, inflammation is the primary driver of relapses and neurological damage, and aggressive suppression of this inflammation with potent therapies can significantly reduce both relapse rates and long-term disability. Early intervention prevents the accumulation of irreversible axonal and neuronal damage, which is the key factor in driving progression to SPMS. Clinical trials have demonstrated that early, aggressive treatment reduces the risk of sustained disability accumulation in RRMS patients, thereby delaying or potentially preventing the transition to SPMS [61]. Moreover, data suggest that patients treated early with highly effective therapies experience better long-term outcomes, including preserved cognitive function, reduced brain atrophy, and improved mobility, all contributing to a better quality of life [62,63]. Real-world evidence further supports that starting with these therapies from the outset leads to more favorable long-term outcomes compared to escalation strategies, where the disease may progress silently before more effective treatments are introduced. The “window of opportunity” to prevent long-term damage is in the early, relapsing phase, where inflammation is more active and treatable. Therefore, in the absence of any treatment which specifically targets the degenerative aspects of the disease, initiating aggressive therapy early in the disease course is the most effective approach to limiting progression and ensuring better long-term outcomes for patients with MS.

## Future treatment options

### Tyrosine kinase [BTK] inhibitors

Bruton’s Tyrosine Kinase (BTK) is a critical enzyme involved in the activation and signaling of B-cells and innate immune cells, such as macrophages and microglia, which play a key role in the pathogenesis of Multiple Sclerosis [64,65]. BTK is also expressed in neurons and astrocytes, though notably absent in oligodendrocytes [66]. BTK inhibitors (BTKi) are small molecules that selectively inhibit BTK activity. Unlike anti-CD20 therapies, which primarily target peripheral B-cells, BTK inhibitors can cross the blood-brain barrier, enabling direct action on microglia and CNS inflammation. This feature is particularly significant for progressive forms of MS, where CNS inflammation and microglial activation are major contributors to disease progression.

In MS, BTK inhibition modulates B-cell activity, including intrathecal follicles, and reduces the inflammatory responses of microglia, both of which are implicated in progressive disease [67]. Several BTK inhibitors are in various stages of clinical development for both relapsing and progressive MS (Table 3).

**Table 3:** Trials of tyrosine kinase inhibitors in MS.

BTK Inhibitor	Type of MS	Mechanism of Action [MOA]	Phase of Study	Latest Available Data Results

Tolebrutinib	RRMS/nSPMS/PPMS	Irreversible BTK inhibition, targets B-cells and microglia with CNS penetration	Phase 3 (RRMS, nSPMS, PPMS)	RRMS: No difference in ARR between tolebrutinib and teriflunomide, 29%risk reduction in CDW [69]
				nSPMS: 31 ​% reduction in CDW [68]
Fenebrutinib	RRMS/PPMS	Reversible BTK inhibition, targets B-cells and microglia	Phase 3 (RRMS, PPMS)	Reduction in new brain lesions in relapsing MS [phase 2]. Early brain penetration data suggests impact on progressive MS [71]
Evobrutinib	RRMS	Reversible BTK inhibition, primarily B-cell focused	Phase 3 (RRMS)	Did not reduce relapse rates compared to teriflunomide [72]
Remibrutinib	RRMS	Reversible BTK inhibition, focus on B-cells and microglia	Phase 3 (RRMS)	Ongoing, preliminary data shows potential lower liver enzyme elevation [73]
Orelabrutinib	RRMS	Irreversible BTK inhibition, strong CNS penetration, modulates microglia	Phase 2 (RRMS)	Ongoing, preliminary data suggests strong CNS penetration with good safety profile [74]

nSPMS (Non relapsing SPMS).

#### Tolebrutinib

Tolebrutinib has demonstrated more encouraging results, especially for non-relapsing forms of MS. In the HERCULES Phase III trial [68], Tolebrutinib reduced confirmed disability progression by 31 ​% compared to placebo in patients with non-relapsing secondary progressive MS. Notably, 10 ​% of patients showed confirmed disability improvement, double the rate observed in the placebo group. However, in the GEMINI trials [69] for relapsing MS, tolebrutinib did not significantly reduce ARR compared to teriflunomide, though it did reduce the risk of disability worsening by 29 ​%. This suggests that tolebrutinib may be particularly effective in addressing smoldering inflammation which is a hallmark of progressive MS. While the efficacy in progressive MS is promising, safety concerns have arisen, particularly related to elevated liver enzymes, with 4.1 ​% of patients experiencing this side effect compared to 1.6 ​% in the placebo group [68]. Tolebrutinib is also being tested in the PPMS population.

#### Fenebrutinib

Fenebrutinib is a reversible BTK inhibitor with broad activity on both B-cells and microglia, making it a strong candidate for progressive forms of MS. The FENtrepid Phase III trial [70] is currently testing fenebrutinib in PPMS and comparing its efficacy against ocrelizumab, the only approved disease-modifying therapy for PPMS. In earlier studies, including the FENopta Phase II trial, [71], fenebrutinib nearly completely suppressed disease activity, as evidenced by a reduction in MRI lesions and halted disability progression over one year of treatment. Its ability to modulate both peripheral and central immune responses may give it an advantage in managing progressive MS, where microglial activation is a major contributor to neurodegeneration.

### Masitinib

Masitinib is a selective tyrosine kinase inhibitor currently under investigation for the treatment of progressive forms of multiple sclerosis. The mechanism of action of masitinib involves the inhibition of immune cells, particularly mast cells and macrophages, which play critical roles in neuroinflammation, and subsequent tissue damage associated with MS [75].

After a previous pilot study suggesting a possible benefit in progressive patients a phase 2b/3 study, masitinib administered at a dosage of 4.5 ​mg/kg/day demonstrated significant efficacy in slowing disability progression compared to placebo [47]. Specifically, the study reported a 42 ​% reduction in the risk of first disability progression and a 37 ​% reduction in the risk of confirmed disability progression. Additionally, masitinib decreased the likelihood of patients reaching a critical disability level defined as an EDSS of 7.0 [47,76]. Building on these promising results, an ongoing Phase 3 trial aims to replicate the efficacy and safety findings in a larger cohort of patients [77]. The outcomes of this trial could provide further evidence for the therapeutic potential of masitinib in managing progressive MS.

### Ibudilast

Ibudilast a phosphodiesterase inhibitor with additional inhibitory effects on macrophage inhibitory factor and Toll-like receptor has not shown efficacy in RRMS but has had promising results in trials on PMS. SPRINT-MS [78] was a phase 2, placebo-controlled, multicenter, randomized trial to evaluate the efficacy and safety of ibudilast at a dose between 60 and 100 ​mg per day. The primary endpoint, whole brain atrophy as measured by brain parenchymal fraction, was reduced by 48 ​% with ibudilast treatment over 96 weeks [79]. Decline in disability progression was similar in the two trial groups. Adverse events more frequent in treatment group than placebo were gastrointestinal symptoms [nausea, diarrhea, abdominal pain, and vomiting] and depression. There was no meaningful difference in the rates or types of infections between the trial groups.

### Foralumab

A human anti-CD3 monoclonal antibody with intranasal administration, was previously found to not only suppress the activity of inflammatory T-cells, but also to increase that of regulatory T-cells in healthy subjects. It has shown promising results in small studies in patients with SPMS with regards to clinical function and microglial activation seen by PET scan [80]. It is currently being studied in a randomized, double-blind placebo-controlled, multicenter dose-ranging study in patients with nSPMS [81]. The primary endpoint will be change in microglial activation on PET at 3 months [81].

### Frexalimab

Frexalimab, another innovative monoclonal antibody, targets the upstream biology of MS by blocking the CD40/CD40L co-stimulatory pathway, which is important for immune cell crosstalk that affects activation of adaptive and innate immune cells without causing lymphocyte depletion [82]. In the Phase 2 trial in participants with relapsing MS, frexalimab showed favorable safety and efficacy and reduction in new gadolinium-enhancing lesions [82]. The efficacy and safety of frexalimab in nSPMS is being studied in a Phase 3 trial –FREVIVA [83].

### Vidofludimus calcium

Vidofludimus calcium is a novel therapeutic agent currently under investigation for the treatment of both relapsing-remitting multiple sclerosis and progressive forms of multiple sclerosis. It is a potent inhibitor of dihydroorotate dehydrogenase (DHODH), with studies indicating that vidofludimus is significantly more effective in inhibiting DHODH oxidation by human DHODH than teriflunomide [84]. This increased potency may contribute to its efficacy in managing MS.

In addition to its role as a DHODH inhibitor, vidofludimus calcium exhibits neuroprotective effects that have not been observed with older therapies. This neuroprotection may be attributed to its activation of the nuclear receptor-related factor 1 (Nurr1), which has demonstrated neuroprotective and anti-neuroinflammatory activities [85]. Nurr1 is an emerging therapeutic target for various neurodegenerative diseases, including MS, Parkinson’s disease, and Alzheimer’s disease [86,87].

The clinical efficacy and safety of vidofludimus calcium are currently being assessed in the Phase II CALIPER trial [88]. Interim analysis results revealed that at 24 weeks, there was reduction in neurofilament light chain (NfL), a marker indicative of neuronal damage and inflammation. Notably, these reductions in NfL levels were consistent across different subtypes of progressive MS, suggesting a potential therapeutic benefit in this population [89].

### CAR-T therapy

Chimeric antigen receptor (CAR) T cells have shown significant therapeutic potential, particularly in targeting malignant B cells in hematologic cancers [90]. This success has prompted exploration of CAR T cells for treating systemic autoimmune diseases caused by autoreactive B cells such as systemic lupus erythematosus (SLE), idiopathic inflammatory [91,92]. Recent studies have also focused on evaluating the effect of anti-CD19 CAR-T cell therapy in CNS autoimmune conditions such as MS, neuromyelitis optica, and myelin oligodendrocyte antibody associated disease [93]. Current treatments rely on long-term immunosuppressive drugs, which fail to fully address the underlying autoimmune processes. CAR T cells targeting the CD19 surface molecule, expressed on a broad range of B cells, offer the potential for deep, long-lasting B-cell depletion and possibly drug-free remission. This approach aims to reprogram immune cells, targeting and depleting pathogenic immune cells to reset the immune system and reduce disease activity.

A notable example is KYV-101, an anti-CD19 CAR-T cell therapy which has received FDA clearance for a Phase 2 trial focusing on patients with treatment-refractory progressive MS [94]. The primary objective of this therapy is to deplete B cells, which are believed to play a significant role in the pathogenesis of MS, particularly in its progressive forms. Early research indicates that CAR-T cells may decreased CSF production of oligoclonal bands and penetrate the CNS more effectively than traditional therapies, such as monoclonal antibodies, which could lead to a more durable remission [93,95].

### Clustered regularly interspaced short palindromic repeats and CRISPR-associated protein 9 (CRISPR-Cas9) technology in MS

CRISPR-Cas9, a highly advanced gene-editing technology, is emerging as a potential therapeutic approach for autoimmune diseases, including MS [96]. By enabling precise modifications of genes implicated in MS pathogenesis, CRISPR-Cas9 holds promise for developing targeted therapies that either modulate immune system activity or promote repair within the CNS. Mechanistically, CRISPR-Cas9 can target genes involved in immune regulation, such as those encoding pro-inflammatory cytokines (e.g., IL-17, IL-23) or molecules essential for T-cell activation, thereby reducing pathological immune responses. Preclinical studies have demonstrated the ability of CRISPR to delete genes related to T-cell autoreactivity, effectively reducing autoreactive T cells and suppressing CNS inflammation in animal models [97,98]. Additionally, CRISPR-mediated targeting of genes like Leucine-rich repeat and Immunoglobulin-like domain-containing protein 1 (LINGO-1) has shown promise in promoting myelin regeneration, while upregulation of neurotrophic factors such as brain-derived neurotrophic factor (BDNF) may offer neuroprotective effects, safeguarding neurons from degeneration [99]. Recent advancements include a genome-wide *in vivo* CRISPR screen in a rat model of MS, which identified key regulators of T-cell migration into the CNS [97]. These findings offer new insights into the immunopathological mechanisms underlying MS and highlight potential avenues for therapeutic intervention.

### Remyelinating strategies

Remyelination strategies in Progressive Multiple Sclerosis (Table 4) focus on repairing damaged myelin sheaths rather than just halting inflammation, aiming to reverse existing damage and distinguishing these approaches from conventional immunosuppressive therapies [100]. One promising avenue is stem cell therapy, which leverages the regenerative potential of stem cells to repair damaged tissues and modulate the immune system. Autologous hematopoietic stem cell transplantation (aHSCT) is a notable method that resets the immune system by eliminating autoreactive immune cells and promoting the regeneration of myelin-producing oligodendrocytes [101,102]. Trials such as the HALT-MS trial [103] have demonstrated significant reductions in disease activity and disability progression in patients with aggressive forms of MS. Another focus is on mesenchymal stem cells (MSCs) which possess neuroprotective, immunomodulatory, and potential remyelinating effects [104]. The MESEMS trial [105], a multinational, randomized, placebo-controlled phase II study, evaluated the safety and efficacy of autologous MSCs in patients with primary and secondary progressive MS. In this trial, which enrolled 144 patients across Europe and Canada, MSC therapy was generally safe, with no significant adverse effects reported. Preliminary MRI data suggested potential neuroprotective benefits, indicated by reduced brain volume loss compared to placebo. However, clinical outcomes such as disability progression did not show significant short-term improvements, highlighting the need for further studies with longer follow-ups to assess functional outcomes and remyelination effects [105].

**Table 4:** Remyelinating treatments for Multiple Sclerosis.

Therapy	Mechanism of Action	Clinical Trials	Outcomes	Challenges

Opicinumab (anti-LINGO-1)	Blocks LINGO-1 protein to promote OPC differentiation and remyelination.	SYNERGY [108]	Mixed results in relapsing MS; did not meet primary endpoints in confirmed disability progression.	Translating remyelination into meaningful clinical benefit remains a challenge in PMS.
		AFFINITY [113]		
Clemastine fumarate	Antihistamine that promotes OPC differentiation and remyelination.	ReBUILD [106]	Improved visual evoked potentials in relapsing MS, trials ongoing in PMS.	Modest remyelination effects; unclear long-term disability benefits in progressive MS.
GSK239512	Histamine H3 receptor antagonist, promotes OPC differentiation and remyelination.	NCT01772199 [114]	Improved MRI outcomes in relapsing MS but no significant clinical benefit in disability reduction.	MRI changes do not always correlate with clinical improvements, especially in PMS.
Mesenchymal stem cells (MSCs)	Stem cells that may provide neuroprotection and promote remyelination.	MESEMS [115]	Demonstrated safety; ongoing trials to assess remyelination and neuroprotection in PMS.	Requires large-scale studies to confirm efficacy and safety in progressive MS.
Ibudilast (MN-166)	Phosphodiesterase inhibitor with potential neuroprotective and remyelinating effects.	SPRINT-MS [79]	Slowed brain atrophy in progressive MS, suggesting neuroprotective effects.	Further trials needed to confirm direct remyelination effects.
Hematopoietic stem cell transplant	Resets the immune system; potential for endogenous remyelination through immune modulation.	HALT-MS trial [103]	Primarily used for aggressive relapsing MS, with possible benefits in PMS through immune reset.	High-risk procedure; neuroprotective and remyelination effects still under investigation.

High-throughput screening identified several FDA-approved anti-muscarinic drugs, notably clemastine, that promote Oligodendrocyte Precursor Cells (OPC) differentiation and remyelination, with clemastine showing improvements in VEP latency and neurofilament levels in MS clinical trials [106]. While clemastine advances in a phase 3 trial, other compounds have produced mixed results. For example, biotin and opicinumab showed early potential but failed to demonstrate significant benefit in later-stage trials, and domperidone increased prolactin without impacting disease progression [[107], [108], [109]]. Additional preclinical candidates targeting pathways such as Notch1 (benztropine), HDAC2 (theophylline), and ERK1/2 (miconazole) may support OPC maturation and remyelination, though further clinical validation is needed [[110], [111], [112]].

Overall, remyelination strategies, particularly stem cell therapies, present a promising avenue for treating progressive MS, but more research is necessary to fully understand their long-term effects on remyelination and functional outcomes in patients with PMS.

### Artificial intelligence [AI] in progressive MS

The application of AI in the treatment of MS holds significant potential for improving patient outcomes, refining diagnostic accuracy, and optimizing therapeutic strategies. AI-driven technologies, including machine learning and deep learning models, are being developed to predict disease progression, monitor therapeutic efficacy, and inform personalized treatment plans using patient-specific data [116]. This is particularly relevant for progressive MS, where the heterogeneity in disease progression complicates treatment decisions.

AI excels in the analysis of complex datasets, including magnetic resonance imaging, clinical metrics, and biomarkers, by identifying subtle patterns that may not be readily apparent to clinicians [117]. Machine learning algorithms have demonstrated considerable potential in predicting the trajectory of progressive MS through the analysis of large imaging datasets, enabling earlier identification of patients at risk for rapid neurodegeneration [118]. Additionally, AI holds promise in optimizing therapeutic strategies, allowing clinicians to tailor treatments more precisely based on predictive insights.

These AI-driven advancements are poised to revolutionize MS care by enhancing diagnostic precision, enabling personalized medicine, and facilitating predictive models for treatment outcomes. Furthermore, AI may extend to the development of automated tools for remote patient monitoring and support, potentially transforming the management of progressive MS.

### Antiviral agents for progressive MS

The premise that multiple sclerosis (MS) is, in part, triggered by infection with the human herpesvirus Epstein Barr virus (EBV) has triggered multiple studies evaluating if treating the virus could influence MS. Most of these trials are ongoing and focus on antiviral therapy in RRMS patients [119,120]. The EMBOLD Phase 1/2 clinical trial [121] tested ATA188 (an experimental cell therapy targeting EBV) in people with nonactive progressive forms of MS, including secondary progressive MS (SPMS) and primary progressive MS (PPMS). In the Phase 1 portion of the study, 24 patients were treated with ATA188 and were followed for one year. Results were promising, with roughly a third of patients showing notable improvement in their disability. In Phase 2, 6 ​% of patients on ATA188 showed disability improvement, as compared to 16 ​% of those given a placebo. Therefore, further phases of this specific trial will not be pursued but there are multiple ongoing trials with other antiviral agents.

## Conclusions

Advancements in understanding and therapies for progressive multiple sclerosis emphasize several key areas where ongoing research and innovation are poised to make significant impacts. In the coming decade, the approval of the first effective remyelination therapies is anticipated, with the potential to not only slow disease progression but also reverse some of the myelin damage characteristic of progressive MS. Additionally, the emergence of new neuroprotective agents, particularly those targeting mitochondrial dysfunction and oxidative stress, is expected to provide more robust options for limiting neurodegeneration. Gene therapy approaches tailored to patients with specific genetic profiles and refined stem cell therapies may also become viable, offering highly personalized treatment options.

The widespread use of biomarkers and genetic profiling is likely to enable the creation of individualized treatment regimens, maximizing therapeutic efficacy while minimizing adverse effects. Advances in precision medicine could allow for lower doses of medications to be used with fewer side effects, particularly in the delivery of neuroprotective and remyelination agents. Improved early detection of progressive MS and more accurate monitoring tools may facilitate earlier interventions, altering the disease trajectory before significant disability occurs.

As our understanding of MS pathophysiology deepens, combination therapies addressing multiple disease pathways are likely to become standard practice. Global collaborations in MS research could accelerate breakthroughs, with data from diverse populations informing the development of universally effective treatments. Future therapies may also incorporate comprehensive care models that address the holistic needs of patients, enhancing overall well-being alongside disease management. Together, these advancements hold the potential to revolutionize the treatment landscape for progressive MS, offering improved outcomes and enhanced quality of life for affected individuals.

## Author contributions

**Marelisa Albelo-Martinez:** Investigation, Writing- Original draft preparation, Figure creation.

**Syed Rizvi**: Conceptualization, Writing- Reviewing and editing, Supervision.

## Declaration of Generative AI and AI-assisted technologies in the writing process

During the preparation of this work the author[s] used CHATGPT for generating some content ideas, refining language and creating charts. After using this tool/service, the authors reviewed and edited the content as needed and take full responsibility for the content of the publication.

## Declaration of competing interest

The authors declare that they have no known competing financial interests or personal relationships that could have appeared to influence the work reported in this paper.
